# Insulin-Regulated *Srebp-1c* and *Pck1* mRNA Expression in Primary Hepatocytes from Zucker Fatty but Not Lean Rats Is Affected by Feeding Conditions

**DOI:** 10.1371/journal.pone.0021342

**Published:** 2011-06-22

**Authors:** Yan Zhang, Wei Chen, Rui Li, Yang Li, Yuebin Ge, Guoxun Chen

**Affiliations:** 1 Department of Nutrition, University of Tennessee at Knoxville, Knoxville, Tennessee, United States of America; 2 College of Pharmacy, South-Central University for Nationalities, Wuhan, Hubei, China; University of Hong Kong, Hong Kong

## Abstract

Insulin regulates the transcription of genes for hepatic glucose and lipid metabolism. We hypothesized that this action may be impaired in hepatocytes from insulin resistant animals. Primary hepatocytes from insulin sensitive Zucker lean (ZL) and insulin resistant Zucker fatty (ZF) rats in *ad libitum* or after an overnight fasting were isolated, cultured and treated with insulin and other compounds for analysis of gene expression using real-time PCR. The mRNA levels of one insulin-induced (*Srebp-1c*) and one insulin-suppressed (*Pck1*) genes in response to insulin, glucagon, and compactin treatments in hepatocytes from *ad libitum* ZL and ZF rats were analyzed. Additionally, the effects of insulin and T1317 on their levels in hepatocytes from *ad libitum* or fasted ZL or ZF rats were compared. The mRNA levels of *Srebp-1c*, *Fas*, and *Scd1*, but not that of *Insr*, *Gck* and *Pck1*, were higher in freshly isolated hepatocytes from *ad libitum* ZF than that from ZL rats. These patterns of *Srebp-1c* and *Pck1* mRNA levels remained in primary hepatocyte cultured *in vitro*. Insulin's ability to regulate *Srebp-1c* and *Pck1* expression was diminished in hepatocytes from *ad libitum* ZF, but not ZL rats. Glucagon or compactin suppressed *Srebp-1c* mRNA expression in lean, but not fatty hepatocytes. However, glucagon induced *Pck1* mRNA expression similarly in hepatocytes from *ad libitum* ZL and ZF rats. Insulin caused the same dose-dependent increase of Akt phosphorylation in hepatocytes from *ad libitum* ZL and ZF rats. It synergized with T1317 to induce *Srebp-1c*, and suppressed *Pck1* mRNA levels in hepatocytes from fasted, but not that from *ad libitum* ZF rats. We demonstrated that insulin was unable to regulate its downstream genes' mRNA expression in hepatocytes from *ad libitum* ZF rats. This impairment can be partially restored in hepatocytes from ZF rats after an overnight fasting, a phenomenon that deserves further investigation.

## Introduction

The increased rate of metabolic diseases, such as obesity, diabetes and cardiovascular disease, has become a major public health concern [1], [2]. The common characteristic of human obesity and type 2 diabetes is insulin resistance [3]. Liver plays a critical role in mediating glucose and lipid homeostasis regulated by hormones and nutrients. Factors derived from adipose tissues, such as free fatty acid [4], adipokines [5] and inflammatory cytokines [6] have been proposed to be responsible for the hepatic insulin resistance.

In liver and hepatocytes, insulin regulates the expression of a variety of genes responsible for glycolysis, glycogenesis and lipogenesis, and inhibits gluconeogenesis [7]. This insulin-regulated hepatic gene expression, at least in part, is responsible for glucose and lipid homeostasis [8], [9]. When liver is insulin sensitive, insulin induces glycolysis, lipogenesis and suppresses gluconeogenesis in hepatocytes. For hepatic glucose metabolism, insulin increases the expression of glucokinase gene (*Gck*) [10], [11], the enzyme responsible for the first step of hepatic glycolysis. It suppresses the expression of the cytosolic form of phosphoenolpyruvate carboxykinase (*Pck1*) [12] and glucose 6-phosphatase catalytic subunit (*G6pc*) [7], the first and last steps of gluconeogenesis, respectively. For hepatic lipid metabolism, insulin increases the expression levels of sterol regulatory element binding protein 1c gene (*Srebp-1c*) [13], a member of sterol regulatory element-binding proteins (SREBPs) which are critical transcription activators for hepatic cholesterol and fatty acid biosynthesis, and their homeostasis [14]. In liver of SREBP-1c deleted mice [15], the fasting-refeeding cycle no longer appropriately regulated the expression levels of critical lipogenic genes such as fatty acid synthase (*Fas*) and stearoyl-CoA desaturase 1 (*Scd1*) [16], [17]. When liver is insulin resistant, insulin no longer suppresses gluconeogenesis, but still stimulates lipogenesis, creating a vicious cycle that aggravates insulin resistance and ultimately contributes to the onset of overt diabetes. The co-existence of hepatic insulin resistance (elevated gluconeogenesis) and sensitivity (elevated lipogenesis) at gene expression level has been observed in rodent diabetic models [3], [8]. However, the mechanism of this co-existence of insulin sensitivity and resistance has not been revealed [18].

The binding of insulin to its receptor initiates a cascade of signal transduction events that lead to metabolic changes in its target tissues [19]. The two well studied pathways activated by insulin stimulation are the phosphatidylinositol 3-kinase (PI3K)–AKT/protein kinase B (PKB) pathway [20] and mitogen-activated protein kinase (MAPK) pathway [21]. Recently, insulin regulated *Srebp-1c* expression has been shown to be mediated by atypical protein kinase C (PKC) [22], [23] and mammalian target of rapamycin complex (mTORC) 1 [24]. Elevated activity of PKCζ in Goto–Kakizaki type 2 diabetic rats has been attributed to the excessive expression of hepatic *Srebp-1c* [25]. However, how these signaling components cause the transcriptional changes remains to be elucidated.

Zucker fatty (ZF) rats [26] and its sub strain Zucker diabetic fatty rats [27] have been widely used as rat models for the development of metabolic diseases [28], [29] due to a missense mutation in the extracellular domain of all leptin receptor isoforms [30]–[32]. Insulin resistance in Zucker fatty or diabetic fatty rats has been associated with higher basal insulin secretion caused by increased fuel metabolism in pancreatic beta cells [33], [34]. The defects in pancreatic beta cell gene expression in Zucker diabetic fatty rats [35], [36], but not in obese ZF rats who have normal glycemia [37], have been attributed to the development of diabetes. However, the insulin-regulated gene expression in hepatocytes from these insulin resistant animals has not been studied.

Recently, in an attempt to understand how insulin induces transcription of its responsive gene, we identified insulin responsive elements in the *Srebp-1c* promoter as two liver X receptor (LXR) binding sites and one sterol regulatory element [38]. This suggests that insulin regulates the expression of its responsive genes after it stimulates the synthesis of endogenous agonists for nuclear receptor activation. It has been reported that the hepatic expression of *Srebp-1c* was elevated in liver of Zucker diabetic fatty rats [39]. We hypothesize that insulin-regulated expression of genes involved in glucose and lipid metabolism may be altered in liver of insulin resistant animals. To focus on insulin resistance and obesity, but not diabetes, we analyzed insulin-regulated gene expression in hepatocytes from ZF rats, which have hyperlipidemia, but normal glycemia [37]. Herein, we report the regulation of the mRNA levels of *Srebp-1c* and *Pck1*, two representative insulin-regulated genes, in hepatocytes isolated from Zucker lean (ZL) and ZF rats.

## Materials and Methods

### Reagents

The reagents for primary hepatocyte isolation and culture have been published [40]. Reagents for cDNA synthesis and real-time PCR were obtained from Applied Biosystems (Foster city, CA). Antibodies to phospho-Akt (Thr473) phospho-Akt (Ser473), and total Akt, were obtained from Cell Signaling Technologies (Danvers, MA). All other compounds were purchased from Sigma (Saint Louis, MO) unless described otherwise.

### Animals

Male ZL and ZF rats were bred at UTK or purchased from Harlan Breeders (Indianapolis, IN). Rats were housed in colony cages, and fed a standard rodent diet before isolation of primary hepatocytes. All procedures were approved by the Institutional Animal Care and Use Committee at the University of Tennessee at Knoxville (Protocol number 1642).

### Hepatocyte isolation and treatments

For primary hepatocyte isolation, ZL or ZF rats in *ad libitum* or fasted overnight as indicated in the figure legends were euthanized with carbon dioxide. A catheter was inserted into portal vein and connected to a peristaltic pump with liver perfusion medium and liver digestive buffer (Invitrogen). The inferior vena cava was cut open to allow the outflow of the media at flow rate of 10 ml/min. After completion of the digestion, livers were excised from the rat and put into a tissue culture plate containing liver digest buffer for removing connection tissues and allowing the release of hepatocytes. Medium containing hepatocytes were filtered through a cell strainer and spun at 50 g for 3 minutes. The cell pellets were washed twice with DMEM containing 5% fetal bovine serum, 100 units/ml sodium penicillin, and 100 µg/ml streptomycin sulfate. After wash, the isolated hepatocytes were plated onto 60-mm collagen type I coated dishes (2 to 3 million cells/dish) and incubated in 4 ml of the same medium at 37°C and 5% CO2. After incubation for 3–4 hours, the attached cells were washed once with 4 ml of PBS, and incubated in medium A (medium 199 with 100 nM dexamethasone, 100 nM 3,3′,5-triiodo-L-thyronine (T3), 100 units/ml penicillin, and 100 µg/ml streptomycin sulfate) containing 1 nM insulin for 14–16 hours until being used for the indicated experiments. For the treatments, primary hepatocytes were washed once with 3 ml of PBS and then incubated in 2 ml of medium A containing indicated reagents for indicated time as shown in the figure legends.

### RNA extraction and Quantitative Real-Time PCR

Methods for preparation and analysis of RNA were described previously [41]. The real time PCR primer sets for detecting *Fas* (from Dr. Bruce Spigelman's group), *Gck*, *Pck1*, and *Srebp-1*c [41] have been published. The primer sets for *Insr* (forward 5′- CTGGAGAACTGCTCGGTCATT-3′, and reverse 5′-GGCCATAGACACGGAAAAGAAG-3′), and *Scd1* (forward 5′- AAGATATCCACGACCCCAGCTA-3′, and reverse 5′- TGCAGCAGGGCCATGAG-3′) were designed using Primer Express software (Applied Biosystems). The gene expression level was normalized to that of 36B4 unless described otherwise. Data were presented as either the fold induction calculated from the ΔΔCt values [40] or the difference of the cycle threshold (ΔCt) numbers between the experimental gene and 36B4, the invariable control gene [41].

### Immunoblot analysis

After indicated treatments in figure legends, primary hepatocytes in a 60 mm dish were washed once with 3 ml PBS and scrapped from the dish in 400 µl of whole-cell lysis buffer (1% Triton X-100, 10% glycerol, 1.0% IGEPAL CA-630, 50 mM Hepes, 100 mM NaF, 10 mM EDTA, 5 mM Sodium orthovanadate, 1.9 mg/ml aprotinin, 5 µg/ml leupeptin, 1 mM Benzamide, 2.5 mM DMSF, pH 8.0). The lysates were allowed to sit on ice for at least 20 minutes before subjected to 20000× g centrifugation for 20 minutes. The protein content in the supernatant was determined with PIERCE BCA protein assay kit (Rockford, IL). Proteins (30 µg/lane) in whole cell lysates were separated on SDS/PAGE, transferred to BIO-RAD Immun-Blot PVDF membrane (Hercules, CA), and detected with primary antibodies according to the protocols provided by the manufacturers. Bound primary antibodies were visualized by chemiluminescence (ECL Western Blotting Substrate; Thermo Scientific) using a 1∶5,000 dilution of goat anti-rabbit IgG (Upstate) conjugated to horseradish peroxidase. Filters were exposed to X-ray films (Phenix Research Products, Candler, NC) for protein band detection.

### Statistics

Data were presented as means ± SD. The number of experiments represented the independent experiments using hepatocytes isolated from different animals on different days. Levene's test was used to determine homogeneity of variance among groups using SPSS 19.0 statistical software and where necessary natural log transformation was performed before analysis. Multiple comparisons were analyzed by one-way ANOVA. The Independent-Samples T-Test was used to compare two conditions. Differences were considered statistically significant at *P*<0.05.

## Results

### Elevated mRNA levels of *Srebp-1c*, *Fas*, *Scd1*, but not *Insr*, *Gck* and *Pck1* in isolated primary hepatocytes from *ad libitum* ZF rats

**Figure 1:**
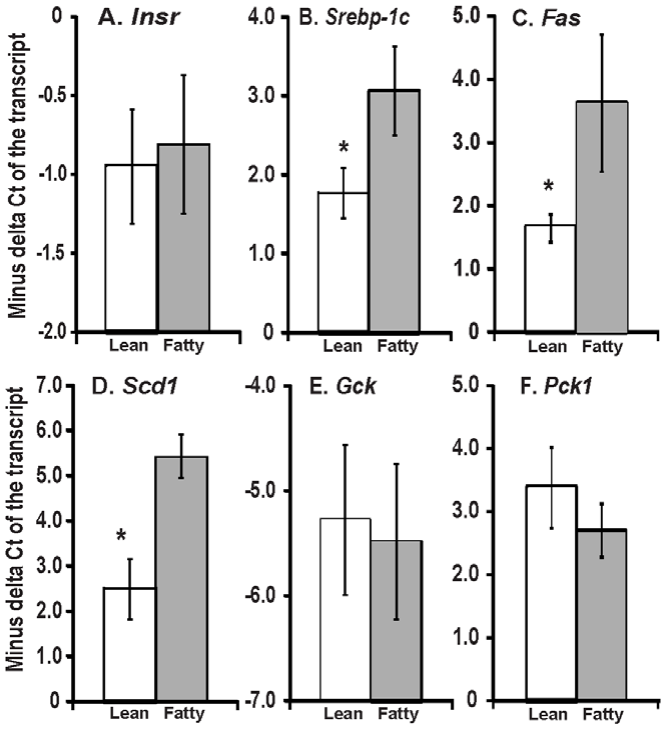
The mRNA levels of *Insr* (A), *Srebp-1c* (B), *Fas* (C), *Scd1* (D), *Gck* (E), and *Pck1* (F) in freshly isolated hepatocytes from *ad libitum* Zucker lean and fatty rats. The primary hepatocytes were isolated from Zucker lean and fatty rats in *ad lib*. The total RNA was extracted and subjected to real-time PCR analysis. Results were presented as means ± SD of −ΔCt (against 36B4) from five different hepatocyte isolations from lean or fatty rats (* all *P*<0.05, for comparing the −ΔCt values of the indicated transcripts in hepatocytes from lean rats with those from fatty rats using independent-samples t test).

To analyze the mRNA levels of hepatic insulin-regulated genes, primary hepatocytes were isolated from ZL and ZF rats in *ad libitum* condition. The mRNA levels of the representative control and insulin-regulated genes involved in hepatic glucose and lipid metabolism were subjected to real-time PCR analysis as shown in Figure 1. The mRNA level of insulin receptor gene (Fig. 1A, *Insr*) in freshly isolated primary hepatocytes of ZF rats was not significantly different from that of ZL rats. The mRNA levels of three lipogenic genes, *Srebp-1c* (Fig. 1B), *Fas* (Fig. 1C) and S*cd1* (Fig. 1D) in the freshly isolated primary hepatocytes of ZF rats were significantly higher than those of ZL rats. On the other hand, the mRNA levels of two genes for glucose metabolism, *Gck* (Fig. 1E) and *Pck1* (Fig. 1F), in the freshly isolated primary hepatocytes of ZF rats were similar to those of ZL rats. All these results demonstrated that ZF rat hepatocytes had significantly higher mRNA levels of *Srebp-1c*, *Fas*, and *Scd1* than ZL rat hepatocytes did, but not that of *Insr*, *Gck*, and *Pck1*.

### The changes of *Srebp-1c* and *Pck1* mRNA levels in response to insulin stimulation were diminished in hepatocytes from *ad libitum* ZF rats

**Figure 2:**
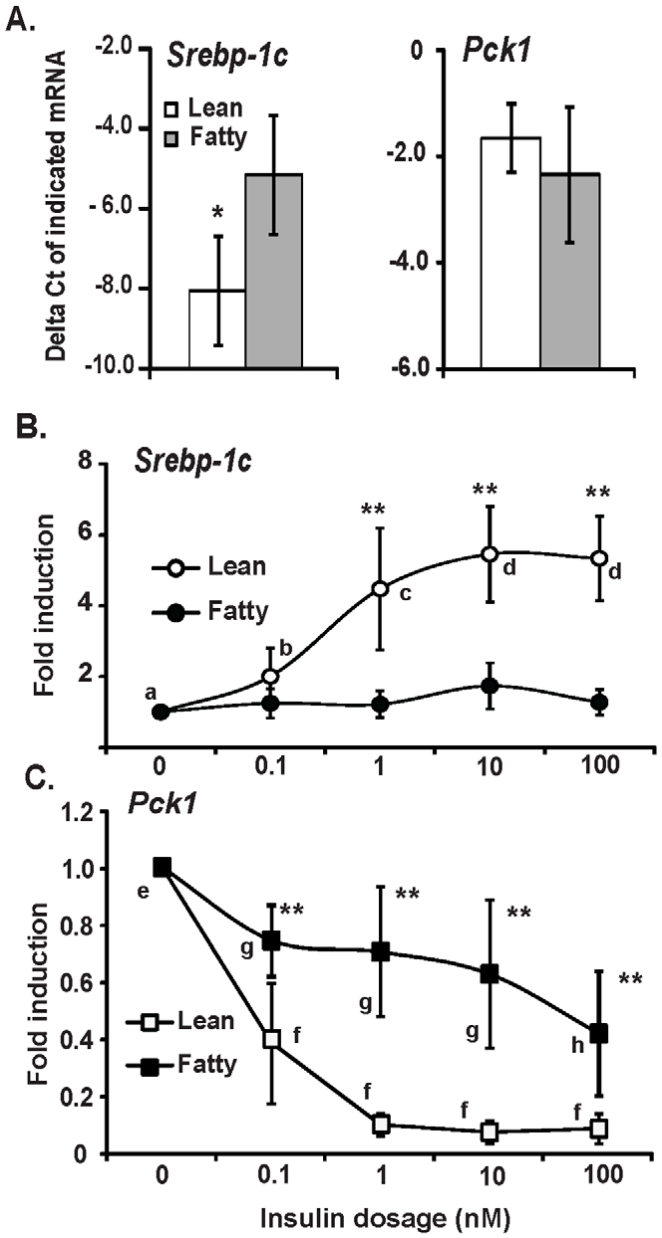
The maintenance of *Srebp-1c* and *Pck1* mRNA expression patterns in primary hepatocyte culture (A), and the responses of *Srebp-1c* (B) and *Pck1* (C) expression to insulin in cultured hepatocytes from *ad libitum* Zucker lean or fatty rats. Primary hepatocytes were isolated and pre-treated as described in Materials and Methods before they were incubated in medium A without or with increasing concentrations of insulin (0.01 to 100 nM) for 6 hours. Total RNA was extracted and subjected to real-time PCR analysis. **A**. The expression levels of *Srebp-1c* and *Pck1* in control hepatocytes were expressed as −ΔCt. **B** and **C**. The expression levels of *Srebp-1c* (**B**) and *Pck1* (**C**) in hepatocytes treated with vehicle control was arbitrarily assigned a value of one for its corresponding cell type. Results were plotted as fold induction and presented as means ± SD of five independent hepatocyte isolations for both lean and fatty rats (n = 5 for hepatocyte isolations; * for comparing the −ΔCt values of lean hepatocytes with fatty hepatocytes using independent-samples t test; ** for comparing fold induction values of the indicated transcripts in lean hepatocytes with that in fatty hepatocytes at indicated insulin concentrations using independent-samples t test; for *Srebp-1c*, c>b/a, d>a, using one-way ANOVA; for *Pck1*, e>f, e>h, using one-way ANOVA; all *P*<0.05,).

To investigate insulin effects on gene expression in hepatocytes from ZL and ZF rats, we examined the regulation of the mRNA levels of two representative genes, one insulin-induced gene (*Srebp-1c*) and one insulin-suppressed (*Pck1*) gene, which are robustly regulated by insulin at transcriptional level in liver and primary hepatocytes [24]. Figure 2A shows the −ΔCt numbers of *Srebp-1c* and *Pck1* of the control groups in lean and fatty hepatocytes after the overnight pretreatment. The *Srebp-1c* mRNA level in fatty hepatocytes was higher than that in lean hepatocytes (−5.2±1.5 *vs* −8.1±1.4). However, *Pck1* mRNA in lean hepatocytes was not significantly different from that in fatty hepatocytes (−2.3±1.3 *vs* −1.7±0.7). These results demonstrated that the primary lean and fatty hepatocytes after overnight pretreatment in culture still maintained similar expression patterns as the freshly isolated hepatocytes did as shown in Figure 1.

To compare the mRNA levels of *Srebp-1c* and *Pck1* in lean and fatty hepatocytes after insulin stimulation, their expression levels in hepatocytes incubated in increasing concentrations of insulin were determined as shown in Figure 2 B and C. Figure 2B shows that the *Srebp-1c* mRNA level in hepatocytes from *ad libitum* ZL rats was significantly induced by insulin at the lowest concentration tested (0.1 nM). However, insulin failed to induce *Srebp-1c* mRNA expression in hepatocytes from *ad libitum* fatty rats. Figure 2C shows that insulin as low as 0.1 nM was sufficient to significantly reduce the *Pck1* mRNA level in lean hepatocytes (0.4±0.23-fold) with further suppression at higher insulin concentrations (Fig. 2C). In contrast, no significant reduction of *Pck1* expression can be observed until insulin reached 100 nM in fatty hepatocytes. The *Pck1* mRNA level in fatty hepatocytes at any insulin concentration tested was significantly higher than that in lean hepatocytes at the corresponding insulin dosage. All these results demonstrated that insulin-mediated regulation of *Srebp-1c* and *Pck1* mRNA expression were significantly diminished or impaired in hepatocytes from fatty rats in *ad libitum*.

### The primary hepatocytes from *ad libitum* ZF rats lost glucagon-suppressed *Srebp-1c* mRNA expression, but retained the glucagon-induced *Pck1* mRNA expression

**Figure 3:**
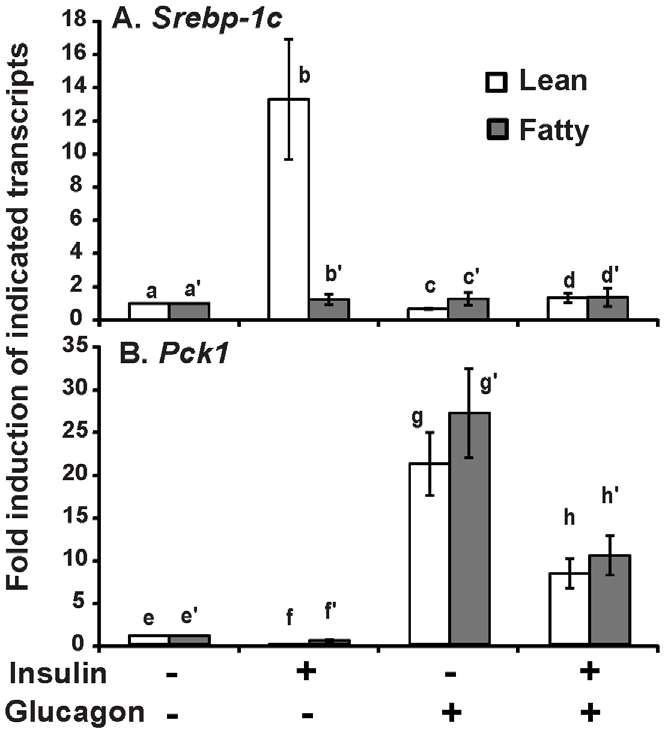
The effects of insulin and glucagon on the mRNA levels of *Srebp-1c* (A) and *Pck1* (B) in hepatocytes from *ad libitum* Zucker lean and fatty rats. Primary hepatocytes after pretreatment as described in Materials and Methods were incubated in medium A without or with 10 nM glucagon in the absence or presence of 100 nM insulin for 6 hours. Total RNA was extracted and subjected to real-time PCR analysis. The expression level of the indicated transcripts in the control hepatocytes (lean or fatty) was arbitrarily assigned a value of one for its corresponding cell type. Results were plotted as fold induction and presented as means ± SD of five independent hepatocyte isolations for both lean and fatty rats (n = 5 for hepatocyte isolations; for *Srebp-1c*, b>a>c, b>d, b>b′, and c′>c, using one-way ANOVA; for *Pck1*, g>h>e>f, g′>h′>e′>f′, and e′>e, using one-way ANOVA; all *P*<0.05).

To examine the responses of hepatocytes to other hormones, hepatocytes from either lean or fatty rats in *ad libitum* were treated with glucagon, an islet derived hormone antagonizing insulin action [42]. Figure 3A shows that insulin induced the *Srebp-1c* mRNA expression in lean hepatocytes as anticipated. Glucagon at 10 nM reduced basal and 100 nM insulin-induced *Srebp-1c* mRNA expression to 0.7±0.04- and 1.2±0.4-fold of the control value, respectively. However, *Srebp-1c* mRNA expression in fatty hepatocytes was not affected by insulin, glucagon or insulin+glucagon treatment. Figure 3B shows that the *Pck1* mRNA expression in lean hepatocytes was significantly suppressed in the presence of insulin. Glucagon dramatically induced *Pck1* mRNA expression to 21±3.7- and 8.2±1.7-fold of the control value in the absence and presence of insulin, respectively. In fatty hepatocytes, insulin at 100 nM still significantly suppressed *Pck1* mRNA expression. However, the remaining *Pck1* mRNA in fatty hepatocytes was still higher than that in lean hepatocytes (0.4±0.1- *vs* 0.04±0.01- fold of the control value). Despite the diminished response of *Pck1* mRNA expression to insulin-mediated suppression in fatty hepatocytes, glucagon dramatically induced its expression by 27±5.2- and 10.2±2.3-fold of the control value in the absence or presence of insulin, respectively. The induction folds of *Pck1* mRNA mediated by glucagon treatment in fatty hepatocytes were not significantly different from those in lean hepatocytes without or with insulin. These results again demonstrated impairment of insulin-regulated *Srebp-1c* and *Pck1* mRNA expression in hepatocytes from ZF rats in *ad libitum*. However, only the glucagon-mediated reduction of *Srebp1-c* mRNA expression, but not induction of *Pck1*, was diminished in the same cells. In addition, the presence of insulin significantly attenuated glucagon-induced *Pck1* mRNA expression in hepatocytes derived from either lean or fatty rats in *ad libitum*.

### Blocking *de novo* cholesterol biosynthesis suppressed basal and insulin-induced *Srebp-1c* mRNA expression in hepatocytes from ZL, but not ZF rats in *ad libitum*

**Figure 4:**
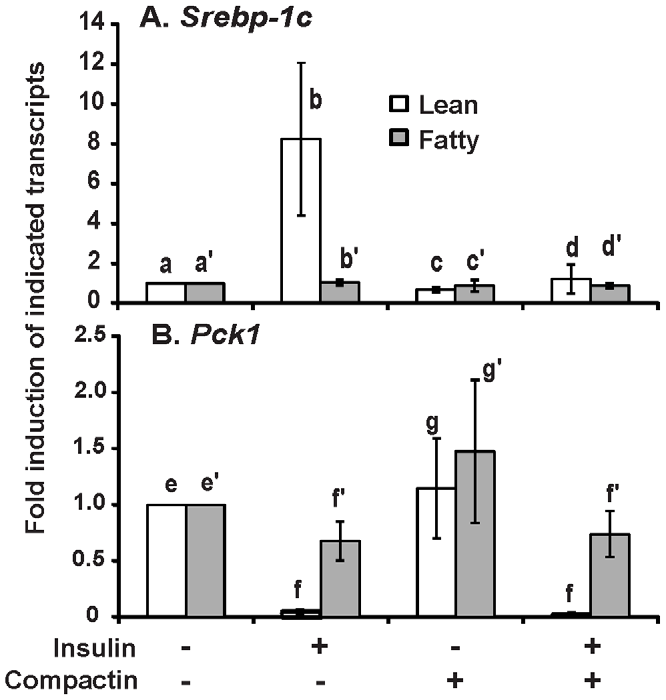
The effects of compactin and insulin on the mRNA levels of *Srebp-1c* (A) and *Pck1* (B) in hepatocytes from *ad libitum* Zucker lean and fatty rats. Primary hepatocytes after pretreatment as described in Materials and Methods were incubated in medium A without or with 50 µM compactin in the absence or presence of 1 nM insulin for 6 hours. Total RNA was extracted and subjected to real-time PCR analysis. The expression level of the indicated transcripts in the control hepatocytes (lean or fatty) was arbitrarily assigned a value of one for its corresponding cell type. Results were plotted as fold induction and presented as means ± SD of four independent hepatocyte isolations for both lean and fatty rats (n = 4 for hepatocyte isolations; for *Srebp-1c*, b>a>c, b>d and b>b′, using one-way ANOVA; for *Pck1*, e/g>f, and f′>f, using one-way ANOVA; all *P*<0.05).

To assess the effects of endogenous cholesterol synthesis on the *Srebp-1c* mRNA expression, hepatocytes from ZL and ZF rats in *ad libitum* were treated without or with insulin in the absence or presence of compactin, an inhibitor of 3-hydroxy-3-methylglutaryl-coenzyme A (HMG CoA) reductase, which has been shown to suppress the synthesis of endogenous ligands for LXR activation [43]. Figure 4A shows that insulin induced *Srebp-1c* mRNA expression in lean, but not fatty hepatocytes, supporting results shown in Figures 2 and 3. Compactin at 50 µM was sufficient to significantly suppress both basal and insulin-induced *Srebp-1c* mRNA expression in lean hepatocytes to 0.6±0.1- and 1.1±0.5-fold of the control value, respectively. However, it did not significantly affect *Srebp-1c* mRNA expression in fatty hepatocytes without or with insulin. Compactin did not affect *Pck1* mRNA expression in hepatocytes from either lean or fatty *ad libitum* rats in the absence or presence of insulin. These results demonstrated that compactin treatment only inhibited basal and insulin-induced *Srebp-1c* mRNA expression in lean, but not that of fatty hepatocytes.

### Insulin-induced phosphorylation of Akt was not altered in hepatocytes from ZF rats in *ad libitum*

**Figure 5:**
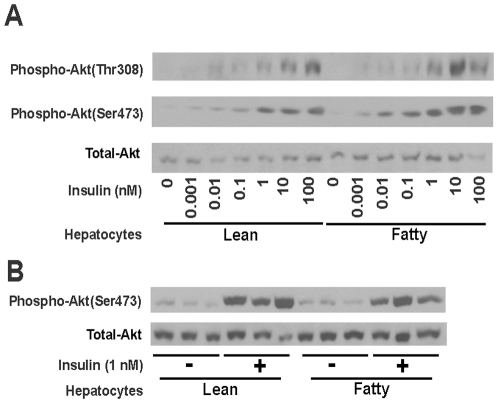
Immunoblot analysis of phospho-Akt and total Akt levels in primary hepatocyes treated with increasing concentrations (A) or 1 nM (B) of insulin. After overnight pretreatment, primary hepatocytes from *ad libitum* ZL and ZF rats were incubated in medium A containing indicated concentrations of insulin for 10 minutes. After which, hepatocytes were washed once with 3 ml PBS and lysed as described in Materials and Methods. Total protein (30 µg/lane) was separated on 8% SDS/PAGE gels, detected by specific primary antibodies as indicated, and visualized by chemiluminescence. **A**. The levels of phoshpo-Akt(Thr308), phosphor-Akt(Ser473), and total Akt in *ad libitum* lean and fatty hepatocytes treated with increasing concentration of insulin (0 to 100 nM). Graph was the representative of three independent experiments with similar results using hepatocytes isolated from three different *ad libitum* ZL or ZF rats in different days. **B**. The levels of phospho-Akt(Ser473) and total Akt in *ad libitum* lean and fatty hepatocytes treated without or with 1 nM insulin. Graph represented three independent isolations of hepatocytes from different *ad libitum* ZL or ZF rats in different days, which were run side by side.

As the first step to investigate insulin signal transduction pathway in hepatocytes from ZL and ZF rats in *ad libitum*, the levels of phosphorylated and total Akt in them after insulin stimulation for 10 minutes was compared. As shown in Figure 5A, insulin started to induce noticeable phosphorylation of Akt on Threonine 308 (Thr308) and Serine 473 (Ser473) as low as 0.01 and 0.001 nM in hepatocytes from either *ad libitum* ZL or ZF rats, respectively. The phosphorylation of Thr308 and Ser473 in both types of hepatocytes reached plateau at 10 and 1 nM insulin, respectively. There was no obvious difference of Akt phosphorylation on these two sites upon insulin stimulation for 10 minutes in hepatocytes from ZL and ZF rats in *ad libitum*. To exclude the variations of Immunoblot performed in different days, the phosphorylation of Akt on Ser473 in three control groups or 1 nM insulin treatment groups of hepatocytes from either ZL or ZF rats in *ad libitum* were compared side by side. As shown in Figure 5B, the levels of Akt phosphorylation on Ser473 in lean hepatocytes were similar to that in fatty hepatocytes from *ad libitum* rats. There was no difference of total Akt protein levels in hepatocytes from lean and fatty rats without or with insulin. These results indicated that insulin-induced phosphorylation of Akt on Thr308 or Ser473 was not impaired in hepatocytes from *ad libitum* ZF rats.

### Fasting restored the induction of *Srebp-1c*, and suppression of *Pck1* mRNA expression mediated by insulin and T1317 in hepatocytes from ZF rats

**Figure 6:**
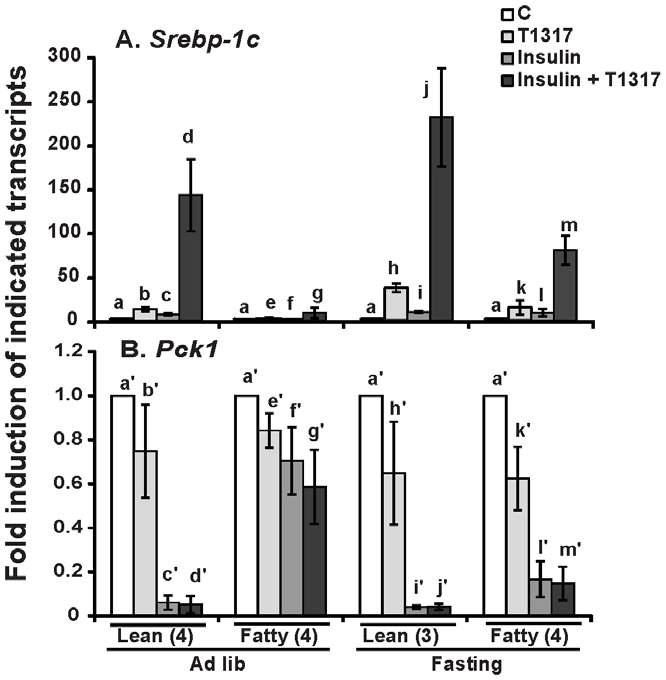
The mRNA levels of *Srebp-1c* (A) and *Pck1* (B) in response to insulin, T1317 and insulin+T1317 treatments in hepatocytes from ZL or ZF rats in *ad libitum* or after fasting for overnight. Primary hepatocytes from rats on different feeding conditions were isolated and pre-treated as described in Materials and Methods. Hepatocytes were incubated in medium A without or with 1 µM T1317 in the absence or presence of 1 nM insulin for 6 hours. Total RNA was extracted and subjected to real-time PCR analysis. The expression level of the indicated transcripts in the control hepatocytes from lean or fatty rats in *ad lib* or overnight fasting was arbitrarily assigned a value of one for its corresponding cell type and feeding condition. Results were plotted as fold induction and presented as means ± SD of indicated numbers (in parenthesis after animal) of independent hepatocyte isolations for both lean and fatty rats (for *Srebp-1c*: d>b>c>a, g>a/f, j>h>i>a, and m>k/l>a, b>e, c>f, d>g, h>b, h>k, j>m, k>e, l>f, and m>g, using one-way ANOVA; for *Pck1*: a′/b′>c′/d′, a′/h′>i′/j′, a′/k′>l′/m′, f′>c′, g′>d′, f′>l′, and g′>m′, using one-way ANOVA; all *P*<0.05).

To explore the effects of feeding condition on the insulin-regulated gene expression, the mRNA levels of *Srebp-1c* and *Pck1* in response to insulin stimulation was analyzed in hepatocytes from ZL and ZF rats in *ad libitum* or after an overnight fasting. Hepatocytes were treated with insulin, T1317 and insulin+T1317, and the expression levels of *Srebp-1c* and *Pck1* mRNA were examined as shown in Figure 6. Figure 6A shows that insulin or T1317 alone induced *Srebp-1c* mRNA expression in hepatocytes from either *ad libitum* or fasted ZL rats. T1317 synergized with insulin to induce *Srebp-1c* mRNA expression dramatically. The induction folds of *Srebp-1c* mRNA mediated by T1317, but not T1317+Insulin, were significantly higher in hepatocytes isolated from fasted lean rats than that that from *ad libitum* lean rats (37.2±5.1- *vs* 12.7±2.6-fold). In hepatocytes isolated from *ad libitum* fatty rats, only insulin+T1317, but not insulin or T1317 significantly induced *Srebp-1c* mRNA expression. In hepatocytes isolated from fasted fatty rats, insulin, T1317 and insulin+T1317 significantly induced *Srebp-1c* mRNA expression to 14.9±8.2-, 8.7±4.1-, and 80±16.6- fold of the control value, respectively. These numbers were significantly higher than those of the corresponding groups in hepatocytes from *ad libitum* rats. The induction folds mediated by T1317 and T1317+insulin in hepatocytes from fasted ZF rats were still lower than those of the corresponding treatments in hepatocytes from fasted ZL rats. These results indicated that overnight fasting partially corrected the impairment of insulin-induced *Srebp-1c* mRNA expression in hepatocytes from ZF rats in *ad libitum*.

Figure 6B shows the *Pck1* mRNA expression levels in hepatocytes from ZL or ZF rats in *ad libitum* or an overnight fasting treated without or with T1317 in the absence or presence of insulin. T1317 at 1 µM did not affect the *Pck1* mRNA expression in hepatocytes from either *ad libitum* or fasted lean or fatty rats in the absence or presence of insulin. Insulin at 1 nM dramatically reduced the expression of *Pck1* mRNA in hepatocytes from *ad libitum* lean rats to the same extent as that in hepatocytes from fasted lean rats. However, it only suppressed *Pck1* mRNA expression in hepatocytes from fasted fatty rats, but not that from *ad libitum* fatty rats. Insulin+T1317 significantly reduced the *Pck1* mRNA expression levels in hepatocytes from *ad libitum* lean, fasted lean and fasted fatty, but not *ad libitum* fatty rats. Insulin and insulin+T1317 significantly reduced the *Pck1* mRNA expression levels in hepatocytes from fasted fatty rats to 0.17±0.08- and 0.15±0.08-fold of the control group value, which are still respectively higher than 0.04±0.008- and 0.04±0.015-fold (the corresponding groups) in hepatocytes from fasted lean rats, indicating partial improvement of insulin action in hepatocytes from fasted fatty rats. These results demonstrated that an overnight fasting of ZF rats restored the insulin-mediated suppression of *Pck1* mRNA expression in their hepatocytes.

## Discussion

In the current study, we observed that the mRNA levels of *Srebp-1c*, *Fas* and *Scd1*, but not that of *Insr*, *Gck* and *Pck1*, were higher in freshly isolated hepatocytes from *ad libitum* ZF rats than those from *ad libitum* ZL rats. It has been reported that ZF rats had hyperinsulinemia, and elevated free fatty acid levels, but normal plasma glucose levels in basal stage or after a glucose load [44]. Elevated expression of hepatic lipogenic genes have been observed in ZF rats [45] and Zucker diabetic fatty rats [39], [46]. Our results matched these original observations. It has been shown that hepatocytes isolated from rats in different nutritional conditions retained their differences in glycogen deposition which is influenced by the nutritional state [47]. The hepatocytes from *ad libitum* fatty rats still retained the expression patterns of *Srebp-1c* and *Pck1* after overnight pre-treatment (Figure 2A). These results indicated that our current experimental settings retained the characteristics of the hepatocytes *in vivo*.

We have shown here that insulin induced *Srebp-1c* and suppressed *Pck1* mRNA expression in hepatocytes from *ad libitum* lean rats. In hepatocytes from *ad libitum* fatty rats, insulin no longer induced *Srebp-1c* mRNA expression at all the concentrations tested. Insulin at 100 nM suppressed *Pck1* mRNA expression in fatty hepatocytes. However, the degree of reduction in fatty hepaotcytes was not comparable to that in lean hepatocytes (Figure 2). This indicates that insulin-suppressed *Pck1* mRNA expression was not completely diminished in fatty hepatocytes, not as insulin-induced *Srebp-1c* was. It has been shown that hepatocytes from the hyperinsulinemic ZF rats had insulin binding equivalent to that of lean littermates and had no reduction of insulin receptor at 10 weeks of age [48]. Therefore, low insulin-binding or receptor expression may not be the reason for the diminished insulin-regulated *Srebp-1c* and *Pck1* mRNA expression. This conclusion is supported by the insulin-mediated Akt phosphorylation, which is similar in hepatocytes isolated from *ad libitum* lean and fatty rats (Figure 5).

Insulin regulates the expression of hepatic genes involved in glucose and lipid metabolism [7], [8]. It induces *Gck* and suppresses *Pck1* [7], both involved in hepatic glucose metabolism. It also induces the expression of *Srebp-1c* mRNA and in turn, increases hepatic fatty acid biosynthesis [13]. If the hyperinsulinemia resulted in the elevated mRNA levels of *Srebp-1c*, *Fas* and *Scd1* in hepatocytes from *ad libitum* fatty rats, the question becomes why it did not cause elevation of *Gck* and reduction of *Pck1* mRNA expression. It seems that the common insulin signaling pathways branched at some points that will specifically determine the responsiveness of a gene to insulin in a certain condition. Indeed, bifurcation of insulin pathway at mTORC1 step has been observed in rat liver which separated the insulin-derived signals responsible for elevation of *Srebp-1c* mRNA from those for reduction of *Pck1* mRNA [24]. Whether any branching point is responsible for the phenomenon observed here remains to be investigated.

The diminished regulation of mRNA levels of *Srebp-1c* and *Pck1* in response to insulin suggested that hepatocytes from *ad libitum* fatty rats might have lost responses to other hormones. Glucagon, a pancreatic hormone antagonizing insulin action [42], has been shown to inhibit *Srebp-1c* and induce *Pck1* mRNA expression in hepatocytes in the absence or presence of insulin [49]. Therefore, glucagon was used to treat hepatocytes from *ad libitum* lean or fatty rats. Glucagon inhibited basal and insulin-induced *Srebp-1c* mRNA expression in lean, but not in fatty hepatocytes from *ad libitum* rats. When the *Pck1* mRNA expression was analyzed, glucagon induced its expression in both lean and fatty hepatocytes to the same extent without or with insulin. These results demonstrated that in fatty hepatocytes, *Pck1* mRNA expression was still responsive to glucagon stimulation. It is noteworthy that insulin attenuated glucagon-mediated induction of *Pck1* mRNA expression in fatty hepatocytes to the same degree as that in lean hepatocytes (Figure 3B). It appears that part of insulin signaling system responsible for regulation of *Srebp-1c* and *Pck1* mRNA expression was impaired in fatty hepatocytes, whereas other parts responsible for attenuation of glucagon action probably remained unchanged. The results obtained from insulin dose-response curves of Akt phosphorylation (Figure 5) supported this conclusion. Insulin dose-dependently phosphorylated Akt on Thr308 and Ser473 to the same extent in hepatocytes from *ad libitum* ZL or ZF rats. These results indicated that activation of Akt by insulin remains the same in lean and fatty hepatocytes. Components of insulin signal transduction pathways other than Akt may be responsible for the impaired response of *Srebp-1c* mRNA expression to insulin in fatty hepatocytes. It has been shown that elevation of PKCζ activity contributed to the increased hepatic *Srebp-1c* expression in type 2 diabetic rats [22], [23] [25]. In addition, insulin-induced *Srebp-1c* mRNA expression in primary rat hepatocytes requires mTORC1 [24]. Whether any of these plays a role in the impairment of insulin-mediated induction of *Srebp-1c* mRNA in primary hepatocytes from *ad libitum* fatty rats remains to be investigated.

As insulin induces *Srebp-1c* transcription via activation of liver X receptor, a nuclear receptor activated by cholesterol derivatives [50], the elevation of its mRNA expression in fatty hepatocytes could be caused by the excessive synthesis of endogenous agonists of LXR activation. However, when the endogenous cholesterol biosynthesis was inhibited by HMG CoA reductase inhibitor compactin [43], the *Srebp-1c* mRNA expression in fatty hepatocytes was not suppressed by it as it was in lean hepatocytes (Figure 4). This suggests that the excessive synthesis of endogenous ligands for LXR activation may not be the reason for unresponsiveness of *Srebp-1c* mRNA expression to insulin in fatty hepatocytes. Alternatively, another ligand not derived from cholesterol might have activated LXR. The fact that T1317 alone, a synthetic ligand for LXR activation [51], could not significantly induce *Srebp-1c* mRNA expression in hepatocytes from *ad libitum* fatty rats (Figure 5A) suggests that the transcription complex at *Srebp-1c* promoter in fatty hepatocytes was not sensitive to changes of ligand for LXR activation as it was in lean hepatocytes. It seems that *Srebp-1c* mRNA expression was locked in a stage which no dynamic change was allowed. This might have caused the unresponsiveness of *Srebp-1c* mRNA in fatty hepatocytes to positive and negative regulatory signals derived from insulin and glucagon, respectively. Whether there is any change of the activities of those transcription complexes on *Srebp-1c* and *Pck1* promoter in fatty hepatocytes deserves further investigation.

As caloric restriction has been shown to increase insulin sensitivity in human and animals [52]–[54], the ZL and ZF rats were fasted for overnight and their hepatocytes were isolated for measurement of their responses to insulin and T1317. In hepatocytes from *ad libitum* or fasted lean rats, T1317 synergized with insulin to induce *Srebp-1c* mRNA expression (Figure 6). Only T1317 alone caused higher induction of *Srebp-1c* mRNA expression in hepatocytes from fasted lean rats than that from *ad libitum* lean rats, demonstrating limited effects of fasting on insulin-regulated gene expression in hepatocytes from lean rats. However, in hepatocytes from fasted fatty rats, insulin induced *Srebp-1c* and suppressed *Pck1* mRNA expression as it did in hepatocytes from lean rats. In addition, T1317 also induced *Srebp-1c* mRNA expression in hepatocytes from fasted fatty rats. The *Srebp-1c* mRNA levels induced by insulin+T1317 in hepatocytes from fasted fatty rats were similar to those from *ad libitum* lean rats, significantly lower than those from fasted lean rats, and significantly higher than those from *ad libitum* fatty rats. These results demonstrated the partial restoration of insulin-regulated gene expression in hepatocytes from fasted fatty rats. It has been shown that an overnight fast was sufficient to mobilize fatty acid from adipose tissues of fatty rats [37]. In addition, fasting has been shown to partially restore insulin actions on adipocytes from insulin resistant ZF rats [55]. The restoration of insulin-regulated *Srebp-1c* and *Pck1* mRNA expression in hepatocytes from fasting fatty rat indicates that short term fasting was sufficient to modify insulin action on hepatocytes from fatty rats. The molecular mechanisms that led to the restoration of insulin action in fatty hepatocytes deserve further investigation.

In summary, we have demonstrated that insulin-regulated *Srebp-1c* and *Pck1* mRNA expression was diminished in hepatocytes from *ad libitum* fatty rats. This impairment was not due to any change of Akt phosphorylation by insulin in hepatocytes from ZF rats. This is the first time that the impairment of insulin action was shown at the regulation of mRNA levels in ZF hepatocytes. The fact that a simple overnight fasting partially restored insulin-regulated gene expression in fatty hepatocytes indicates the existence of potential pathway which can reverse the insulin resistance in hepatocytes from Zucker fatty rats. The understanding of the underlying molecular mechanisms will help us to combat metabolic diseases.
